# Is a Higher Body Mass Index a Risk Factor for Developing Antiphospholipid Antibody Syndrome?

**DOI:** 10.7759/cureus.42982

**Published:** 2023-08-05

**Authors:** Eman M Mansory, Atheer M Badawi, Renad Rajab, Asma Abdullah, Mudhawi Alhiniah, Rakan H Alelyani, Jamil Al-Mughales, Hatem M AlAhwal, Ahmed S Barefah

**Affiliations:** 1 Department of Hematology, King Abdulaziz University, Jeddah, SAU; 2 Hematology Research Unit, King Fahd Medical Research Center, Jeddah, SAU; 3 Faculty of Medicine, King Abdulaziz University, Jeddah, SAU; 4 Department of Clinical Laboratories, Diagnostic Immunology Division, King Abdulaziz University, Jeddah, SAU; 5 Department of Clinical Microbiology and Immunology, King Abdulaziz University, Jeddah, SAU

**Keywords:** antiphospholipid antibodies, obesity medicine, clinical immunology, thrombosis, body mass index: bmi

## Abstract

Background: Antiphospholipid antibodies (aPLs) are antibodies directed against components of the cell membrane and can be associated with clinical features or be asymptomatic in 1-5% of the population.

Objective: The objective of this study is to investigate the frequency of aPL positivity based on body mass index (BMI).

Methods: This is a retrospective analysis of all aPL testing done in a tertiary center between 2010 and 2020. The difference between patients with BMI <25, BMI 25-30, and BMI>30 is calculated using chi-square or Fisher's exact test as appropriate for categorical variables and a two-sample t-test for numerical variables. Unadjusted then multivariable logistic regression models were conducted to evaluate the effect of BMI on aPL positivity adjusting for age, thrombosis history, pregnancy complications history, and presence of autoimmune disease. Sex was included as an effect modifier.

Results: Among 312 patients, the outcome (any positive aPL) was detected in 26 (20.8%), 13 (13.0%), and 16 (18.4%) patients with BMI groups: BMI <25, BMI 25-30, and BMI > 30, respectively. A multivariable logistic regression showed that those with BMI 25-30 had a lower risk of aPL positivity when compared to patients with BMI <25 (OR of 0.55 CI 0.25 - 1.14, p=0.116), and patients with BMI >30 also carried a lower risk compared with patients with BMI<25 (OR of 0.76, 95% CI 0.36 - 1.56, p=0.46); these results were not statistically significant.

Interpretation: The results suggest that a higher BMI was not a risk factor for aPL positivity. A better understanding of the complex interactions between antiphospholipid antibodies and obesity should be further investigated.

## Introduction

Antiphospholipid antibody syndrome (APS) is a thrombotic condition that manifests clinically by significantly increasing the risk of thrombotic events, including arterial and venous, as well as pregnancy complications and fetal loss. The disorder is caused by the body's immune system producing abnormal antibodies called antiphospholipid antibodies (aPLs), and the diagnosis is based on clinical criteria in addition to serological presence of these antibodies, including anticardiolipin antibodies, anti-B2-glycoprotein antibodies, and lupus anticoagulant on two occasions 12 weeks apart [1]. The diagnosis can be primary or secondary depending on the presence of other coexisting autoimmune conditions.

Although APS is not a common condition with an estimated prevalence of 40-50 cases per 100,000 persons [2], it constitutes about 20% of all cases of thrombosis in people under the age of 50 [3]. Interestingly though, aPLs (antibodies without clinical symptoms) are not uncommonly encountered (One to five percent of healthy individuals have aPLs) [4].

Overweight and obesity are established risk factors for the development of venous thromboembolism (VTE) and morbidities during pregnancy [5]. Moreover, a study found that patients with both APS and obesity have worse outcomes namely venous thromboembolism (46.6% in patients with body mass index (BMI) >or= 30 vs. 14.2% in patients with BMI<30, P = 0.02) and obstetric events (53.3% in patients with BMI >or= 30 vs. 22.8% in patients with BMI < 30, P = 0.04) [6].

To understand this complex relationship between obesity and APS, a retrospective study looked at the prevalence of obesity in patients with primary APS and found that APS was more frequent in patients with obesity and hypothesized that the elevated inflammatory states in obese patients increased their risk of developing APS [7]. However, the study was limited by including only patients with APS and no control group with asymptomatic patients with aPLs, or patients with negative antibody testing. There have been no other studies examining the relationship between aPL positivity and obesity. Therefore, this study is examining the frequency of aPLs according to BMI regardless of an APS diagnosis. Knowledge of the relationship between BMI and aPL positivity would certainly be of value in evolving our current understanding of aPLs and their implications.

## Materials and methods

Study design and data sources

This is a retrospective observational study looking at data for all aPL testing for any indication done at a tertiary hospital’s laboratory center at King Abdulaziz University Hospital (a total bed capacity of 845) in Jeddah, Saudi Arabia, between the years of 2010 and 2020, complemented with chart review to collect clinical data on patients that had undergone testing. The chart review extracted variables like age, sex, body mass index (BMI), admission diagnosis and comorbidities, use of anticoagulation and type of anticoagulant, thrombosis history and type, pregnancy history (including number of abortions or pregnancy complications), and number of follow-up visits. The data include 414 patients who underwent any aPL testing. Ethical approval for the study was obtained.

Analytic sample

**Figure 1:**
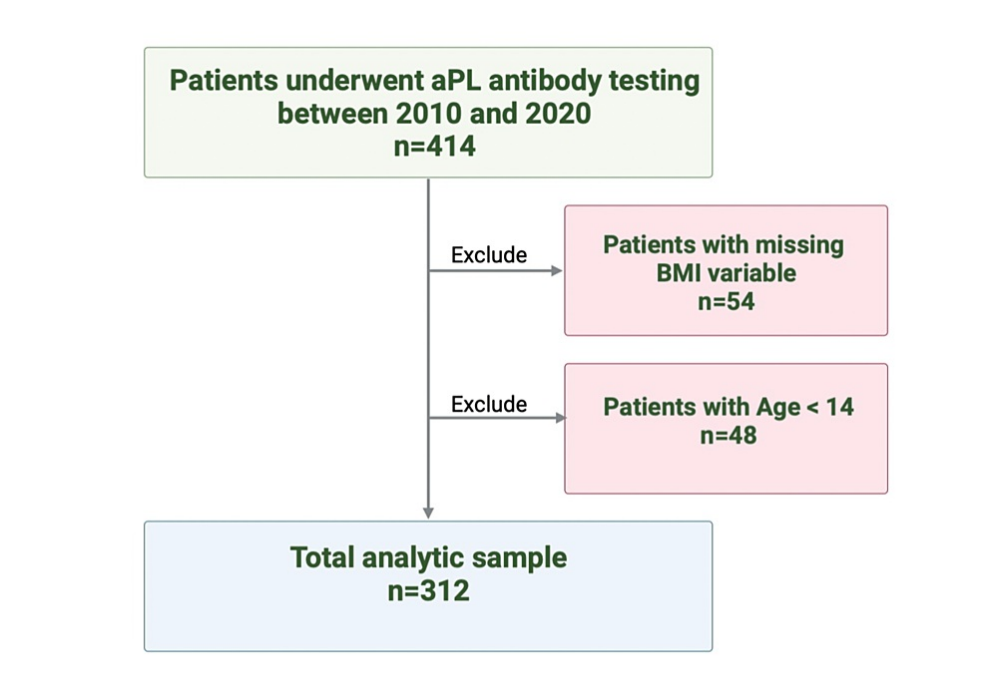
Analytic sample BMI: Body mass index; aPL: antiphospholipid antibody

Patients are included in this study if they are above the age of 14 years (patients are managed by the internal medicine service rather than pediatrics after the age of 14 in this center) and underwent testing for any aPL testing for any indication, patients don’t need to have all three tests done to be included. Patients were excluded if they are younger than 14 years of age, or if their BMI was missing. The final analytic sample consisted of 312 distinct patients (see Figure 1 for details). Of note, there were periods when the reagents for aPL testing were not available, leading to a less-than-expected sample size and the inability to assess the trends in testing. Lupus anticoagulant testing included in the sample was reported to be off anticoagulants to be accepted in the lab.

Study variables

The study’s outcome variable is having a positive result for any of the antiphospholipid antibodies (lupus anticoagulant, anticardiolipin antibodies “IgG or IgM” or B2-glycoprotien “IgG or IgM”) analyzed as a dichotomous variable (any positive result vs negative). The explanatory variable is the patients BMI obtained through chart review that has the measured height and weight of patients done by nursing staff and then BMI calculated using the formula (weight in kilograms/ height in meters squared) and categorized as BMI<25 (normal weight) vs BMI 25-30 (overweight) and BMI>30 (obesity), there were only 12 patients with a BMI under 18.5 (underweight) and so they were not separated in an independent group. Other important variables that can have a confounding effect and will be included in the analysis are age (numerical variable), presence of autoimmune disease (yes/no), presence of thrombosis history; both venous events (deep venous thrombosis, pulmonary embolism, splanchnic thrombosis or cerebral venous thrombosis) and arterial thrombotic events including myocardial infarction or strokes, thrombosis history will be analyzed as a categorical variable (yes/no), and finally, history of pregnancy complications, which include abortion and history of pre-eclampsia/eclampsia (yes/no). These variables were obtained through chart review.

Analysis plan

Characteristics of study patients who met inclusion criteria were described using the mean for continuous variables and proportion for categorical variables. Group differences were compared between patients with BMI <25, BMI 25-30, and BMI>30 using chi-square or Fisher's exact test as appropriate for categorical variables and a two-sample t-test for numerical variables.

Following the above descriptive analysis of the study sample and the different variables, a multivariate logistic regression model will be used to evaluate the effect of BMI on antiphospholipid antibody positivity adjusting for age, thrombosis history, pregnancy complication history, and presence of autoimmune disease. Analysis of the data was conducted using R and R Studio [8,9].

## Results

The study included 312 distinct patients aged 14 and above who had undergone antiphospholipid antibody testing between 2010 and 2020 and had measured BMI on file. The sample was dominated by females: 264 (84.62%) females and 48 (15.38%) males, the age ranged between 14 and 84 years with a mean of 37. The BMI in the patient’s sample ranged between 13.33 and 55.44 with a mean of 29. Thrombosis history, arterial and venous, was positive in 89 patients (28.53%) and pregnancy morbidity history was positive in 92 (34.85%) female patients. Autoimmune disease history was positive in 62 patients (19.87%). There were 55 (17.63%) patients who had at least one of the aPLs positive, and 23 (7.37%) patients who had a documented diagnosis of antiphospholipid antibody syndrome.

There were 125 patients with a normal BMI <25, 100 patients with overweight (BMI 25-30), and 87 patients with obesity (BMI>30). There was no significant difference in sex distribution between the three exposure groups. Moreover, there was no significant difference between the three groups when it comes to the history of autoimmune disease, Thrombosis history, use of anticoagulants, or antiphospholipid positivity. Patients with a higher BMI were found to be slightly older with higher prevalence of pregnancy complications. See full details in Table 1.

**Table 1:** Patients' characteristics according to the BMI level aPL: Antiphospholipid antibody, BMI: body mass index, DOACs: direct oral anticoagulants, LMWH: low-molecular-weight heparin. ^1^SD= Standard deviation. ^2^Missing data: Age in (13.1%). ^3^Any positive aPL includes any patient with a positive antiphospholipid antibody testing including (positive Lupus anticoagulant or positive B2-glycoprotien, or positive anticardiolipin antibody). aPL1 indicates the first set of aPLs done for the patient. ^4^aPL2 indicates the second set of aPLs done for the patient. Group differences were compared using chi-square or Fisher's exact test as appropriate for chaological variables (sex, autoimmune disease, history of thrombosis, pregnancy complications, Any positive aPL1, Any positive aPL2, warfarin, heparin, LMWH, and DOACs) and one-way test for the numerical variable (age).

Variable	BMI 0-25	BMI 25-30	BMI 30-200	p-value
n	125	100	87	
sex = female (%)	110 (88.0)	84 (84.0)	70 (80.5)	0.319
Age (mean (SD))^ 1,2^	36.0 (15.1)	37.6 (12.9)	41.0 (12.5)	0.031
Autoimmune disease = yes (%)	26 (20.8)	20 (20.0)	16 (18.4)	0.910
Thrombosis = yes (%)	32 (25.6)	24 (24.0)	33 (37.9)	0.071
Pregnancy complications = yes (%)	27 (21.6)	35 (35.0)	30 (34.5)	0.044
Antibodies Testing:				
Any Positive aPL1^3^= yes (%)	26 (20.8)	13 (13.0)	16 (18.4)	0.305
Any Positive aPL2^4^= yes (%)	8 ( 6.4)	6 ( 6.0)	10 (11.5)	0.291
Anticoagulants:				
WARFARIN = yes (%)	23 (18.4)	18 (18.0)	21 (24.1)	0.500
HEPARIN THERAP = yes (%)	14 (11.2)	7 ( 7.0)	7 ( 8.0)	0.515
LMWH THERAP = yes (%)	14 (11.2)	8 ( 8.0)	16 (18.4)	0.087
DOAC = yes (%)	1 ( 0.8)	3 ( 3.0)	1 ( 1.1)	0.394

The outcome, any positive antiphospholipid antibody (aPL), was detected in 26 (20.8%), 13 (13.0%), and 16 (18.4%) patients with BMI groups: BMI <25, BMI 25-30, and BMI>30 respectively. The outcome includes any positive result for lupus anticoagulant, anticardiolipin antibody (IgM and IgG), and B2 glycoprotein (IgG and IgM). There was no significant relationship between positive results of aPL and BMI group, and the p-value was 0.305. This was also maintained when the results of each antibody subtype were examined in relation to BMI. For detailed results for aPLs by subtype, please refer to Table 2.

**Table 2:** Results of subtypes of aPLs according to the BMI level aPLs: antiphospholipid antibodies; BMI: body mass index; LAC: lupus anticoagulant; ACA: anticardiolipin antibody; B2G: B2-Glycoprotien.

Variable	BMI 0-25	BMI 25-30	BMI 30-200	p-value
n	125 (%)	100 (%)	87 (%)	
LAC				0.949
Neg	68 (81.9)	56 (86.2)	50 (84.7)	
Mild Positive	14 (16.9)	8 (12.3)	8 (13.6)	
High Positive	1 ( 1.2)	1 ( 1.5)	1 ( 1.7)	
ACA IgG				0.746
0.20	34 (57.6)	22 (50.0)	29 (61.7)	
20-40	15 (25.4)	14 (31.8)	13 (27.7)	
>40	10 (16.9)	8 (18.2)	5 (10.6)	
ACA IgM				0.511
<20	55 (57.3)	40 (59.7)	44 (69.8)	
20-40	19 (19.8)	15 (22.4)	9 (14.3)	
>40	22 (22.9)	12 (17.9)	10 (15.9)	
B2G IgG				0.666
>40	4 (13.3)	6 (27.3)	5 (17.9)	
<20	14 (46.7)	10 (45.5)	15 (53.6)	
20-40	12 (40.0)	6 (27.3)	8 (28.6)	
B2G IgM				0.850
<20	33 (63.5)	24 (63.2)	20 (54.1)	
20-40	13 (25.0)	8 (21.1)	11 (29.7)	
>40	6 (11.5)	6 (15.8)	6 (16.2)	

Unadjusted logistic regression showed that although not significant, those with a BMI of 25 - 30 were less likely to have aPL positivity compared to those with a BMI < 25 (Odds ratio (OR) 0.569, 95% confidence interval (CI) 0.268 - 1.16, p = 0.128). Those with a BMI>30 were similarly as likely to have aPL positivity compared to those with a BMI<25 (OR 0.858, 95% CI 0.422 - 1.70, p = 0.665). Furthermore, examining a multivariable logistic regression model adjusting for age category, history of thrombosis, and history of autoimmune disease also showed a somewhat similar result, with those with a BMI of 25-30 carrying the lower risk of aPL positivity when compared to patients with BMI <25 (OR of 0.550, 95% CI 0.254 - 1.14, p=0.116), patients with BMI>30 also carried a lower risk compared with patients with BMI<25 (OR of 0.762, 95% CI 0.363 - 1.56, p=0.462), these results were not statistically significant. Please see details of univariate and multivariate analysis in Table 3.

**Table 3:** Results of univariant and multivariable logistic regression analysis of the risk of antiphospholipid antibody positivity in patients according to the BMI level BMI: Body mass index; OR: Odds ratio; CI: confidence interval Multivariable logistic regression analysis model was done adjusting for age category, history of thrombosis, and history of autoimmune disease

Variables	Univariate analysis OR (95% CI), p-value	Multivariate analysis OR (95% CI), p-value
BMI < 25	Reference level	Reference level
BMI 25- 30	0.569 (0.268 -1.16), p-value 0.128	0.550 (0.254 - 1.14), p-value 0.116
BMI > 30	0.858 (0.422- 1.70), p-value 0.665	0.762 (0.363 - 1.56), p-value 0.462

A further analysis using sex as an effect modifier since females are at higher risk of developing APS and autoimmune diseases generally showed that in females the difference in odds is less accentuated compared to Males. In females, OR for any positive aPL was lower in patients with overweight or obesity when compared to patients with normal BMI though this was not statistically significant, OR 0.538 (0.227 - 1.20) p= 0.142 for patients with overweight and OR 0.601(0.247 - 1.38) p=0.242 for patients with obesity. On the other hand, for males, the risk for patients with obesity was comparable to patients with normal BMI (reference group) with OR 1.08 (0.190 - 6.40) p= 0.931. Males with overweight carried a non-statistically significant lower risk of aPL positivity with OR 0.367 (0.0442 - 2.50), p-value 0.315. Effect modification was thought to be present as noted through the different coefficients between males and females with significantly wider confidence intervals in males. See Table 4 for detailed results. For both groups (males and females), the model was adjusted for age category, history of thrombosis, and history of autoimmune disease. This result was maintained even when the analysis was done categorizing BMI into two groups only: normal (<25) and high (>25).

**Table 4:** Multivariable logistic regression analysis of the risk of antiphospholipid antibody positivity in patients according to the BMI level and the effect modifier sex BMI: Body mass index; OR: Odds ratio; CI: confidence interval The multivariate logistic regression analysis model was done adjusting for age category, history of thrombosis, and history of autoimmune disease in both males and females.

Variables	Multivariable analysis in females OR (95% CI), p-value	Multivariate analysis in males OR (95% CI), p-value
BMI < 25	Reference level	Reference level
BMI 25- 30	0.538 (0.227 - 1.20), p-value 0.142	0.367 (0.0442 - 2.50), p-value 0.315
BMI > 30	0.601 (0.247 - 1.38), P-value 0.242	1.08 (0.190 - 6.40), p-value 0.931

## Discussion

The key results of this study show a possibly lower risk of aPL positivity in both patients with overweight (BMI 25-30) and patients with obesity (BMI> 30) when compared to patients with BMI<25, though this was not statistically significant. These findings were against our study’s hypothesis and what has been observed in other studies. It is important to note that previous reports were on patients with APS diagnosis only who had a previous venous thrombosis history with no control group [7], and this study included all comers tested for aPLs regardless of indication, and then exposure groups were also adjusted to the confounding effect of thrombosis. Thus, although the results were against our hypothesis, this is not entirely surprising given the population of the study. This is somewhat supported by a study that looked at the difference in characteristics between patients with APS and asymptomatic aPL carriers and found that the differences between the two groups were at least partially dependent on the proportion of coincident vascular risk factors [10].

We also note in this study a significant predominance of females being tested for aPLs which is likely related to the frequent testing of aPLs in patients with a history of pregnancy losses and pregnancy complications. In addition to the fact that females carry a higher risk of autoimmune disease, which might have led to more testing in female patients, given the nature of the study, it is challenging to comment whether there was over or under-testing leading to this observation. Moreover, overweight and obesity were frequent in the sample, which is consistent with the high rates of overweight and obesity in Saudi Arabia [11,12].

A sensitivity analysis looking at aPL results based on patients’ categories whether asymptomatic, had a pregnancy complication, or venous thromboembolism, would be very helpful as a further step to expand on our understanding of the aPLs in different patient populations. In addition, knowledge of indications for testing as well as information on other inflammatory disorders and malignant conditions that might affect aPLs would have further strengthened the analysis.

The aPLs are heterogenous with many factors causing them to be positive as well as medications that can interfere with results, and like many laboratory tests for autoimmune disease, there are no international standards to express the test results in international units and different centers use different testing procedures and thresholds as well as different combinations of antibodies being tested for [13,14]. All of these factors make studies on aPLs face many challenges and very difficult to generalize. This has called for updating the classification guidelines for APS which is currently being developed and will make future studies more standardized [15].

As our understanding of the different aspects of aPLs expands, especially in asymptomatic patients, this study’s advantage is that it is the first study trying to understand the relationship between BMI and risk of aPL positivity, an area of significant knowledge gap. Yet the result of this study needs to be put into the context of being a retrospective study of an unselected population with the inherent limitations of this design. Moreover, the study’s sample was relatively small coming from a tertiary care center, partially due to the unavailability of aPL reagents at some points during the study period, and the exclusion of some patients due to missing data. This was also a single-center study from a governmentally funded institution that serves a population that may carry unique characteristics. Finally, BMI recorded in files might not have been the same at the time of testing, and this might have affected the results.

The results of this study are hypothesis-generating and a primary step toward an enhanced understanding of aPLs, their epidemiology, and their different implications in various clinical scenarios. Further large multicenter studies are certainly needed for more accurate estimates.

## Conclusions

In conclusion, higher BMI was not found to be a significant risk factor for aPL positivity. Certainly, a larger study is needed before drawing any hard conclusions and a better understanding of the complex interactions between antiphospholipid antibodies, vascular risk factors, and obesity should be further investigated.
